# *N*′-Benzoyl-3-hydr­oxy-2-naphthohydrazide

**DOI:** 10.1107/S1600536808012919

**Published:** 2008-05-07

**Authors:** Qi-Feng Liang, Hai-Mei Feng, Feng-Qing Li

**Affiliations:** a Department of Chemistry, Jiaying University, Meizhou 514015, People’s Republic of China; b State Key Laboratory Base of Novel Functional Materials and Preparation Science, Institute of Solid Materials Chemistry, Faculty of Materials Science and Chemical Engineering, Ningbo University, Ningbo 315211, People’s Republic of China; c School of Environmental and Biological Science and Technology, Dalian University of Technology, Dalian 116024, People’s Republic of China

## Abstract

In the title compound, C_18_H_14_N_2_O_3_, the dihedral angle between the planes of the naphthalene and phenyl ring systems is 2.64 (2)°. Mol­ecules are engaged in π–π stacking (mean interplanar distance = 3.339 between naphthalene rings and 3.357 Å between benzene rings )and hydrogen-bonding inter­actions.

## Related literature

For related literature, see: Alexiou *et al.* (2002 ▶); Gaynor *et al.* (2002 ▶); Lah & Pecoraro (1989 ▶); Lehaire *et al.* (2002 ▶); Liu *et al.* (2001 ▶); Saalfrank *et al.* (2001 ▶).
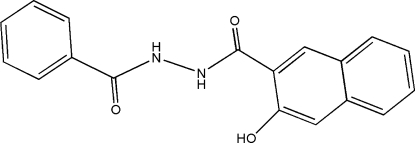


## Experimental

### 

#### Crystal data

C_18_H_14_N_2_O_3_
*M* *_r_* = 306.31
Monoclinic, 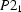

*a* = 4.8049 (10) Å
*b* = 5.0231 (10) Å
*c* = 29.398 (6) Å
β = 91.59 (3)°
*V* = 709.3 (2) Å^3^
*Z* = 2
Mo *K*α radiation
μ = 0.10 mm^−1^
*T* = 273 (2) K
0.35 × 0.24 × 0.14 mm

#### Data collection

Rigaku R-AXIS RAPID diffractometer
Absorption correction: multi-scan (*ABSCOR*; Higashi, 1995 ▶) *T*_min_ = 0.927, *T*_max_ = 0.984
6959 measured reflections
1798 independent reflections
1397 reflections with *I* > 2σ(*I*)
*R*_int_ = 0.044

#### Refinement

*R*[*F* ^2^ > 2σ(*F* ^2^)] = 0.047
*wR*(*F* ^2^) = 0.112
*S* = 1.05
1798 reflections
208 parameters
1 restraint
H-atom parameters constrained
Δρ_max_ = 0.18 e Å^−3^
Δρ_min_ = −0.18 e Å^−3^

### 

Data collection: *RAPID-AUTO* (Rigaku, 1998 ▶); cell refinement: *RAPID-AUTO*; data reduction: *CrystalStructure* (Rigaku/MSC, 2002 ▶); program(s) used to solve structure: *SHELXS97* (Sheldrick, 2008 ▶); program(s) used to refine structure: *SHELXL97* (Sheldrick, 2008 ▶); molecular graphics: *CrystalStructure*; software used to prepare material for publication: *CrystalStructure*.

## Supplementary Material

Crystal structure: contains datablocks global, I. DOI: 10.1107/S1600536808012919/hg2387sup1.cif

Structure factors: contains datablocks I. DOI: 10.1107/S1600536808012919/hg2387Isup2.hkl

Additional supplementary materials:  crystallographic information; 3D view; checkCIF report

## Figures and Tables

**Table 1:** Hydrogen-bond geometry (Å, °)

*D*—H⋯*A*	*D*—H	H⋯*A*	*D*⋯*A*	*D*—H⋯*A*
O1—H1*C*⋯O2^i^	0.82	2.00	2.818 (3)	174
N1—H1*B*⋯O1	0.86	1.96	2.652 (4)	137
N2—H2*B*⋯O3^ii^	0.86	2.09	2.826 (3)	143

Symmetry codes: (i) 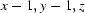; (ii) 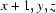.

**Fig. 1.**
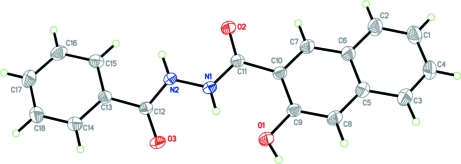
The structure of (I), showing 30% probability displacement ellipsoids and the atom-numbering scheme.

**Fig. 2.**
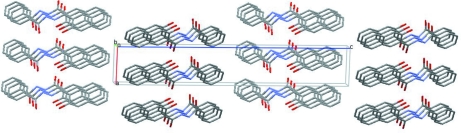
A view of π-π stacking of (I). H atoms have been omitted.

## supplementary crystallographic information

### Comment

Metallacrowns are a new class of metallamacrocycles, which have gained increasing attention over the past decade because of their potentially unique properties (Alexiou *et al.*, 2002; Gaynor *et al.*, 2002; Lah & Pecoraro, 1989; Lehaire *et al.*, 2002; Liu *et al.*, 2001; Saalfrank *et al.*, 2001). These metallacrowns exhibit selective recognition of cations and anions (Saalfrank *et al.*, 2001; Lehaire *et al.*, 2002), can display intramolecular magnetic exchangeinteractions (Liu *et al.*, 2001), and can be used as building blocks for two-dimensional or three-dimensional network structures (Gaynor *et al.*, 2002; Lah & Pecoraro, 1989; Lehaire *et al.*, 2002). The ability to control the generation of metallacrowns with different nuclear numbers, desired structures, and properties is still a substantial challenge. We now report structure of a designed pentadentate ligand, 3-hydroxy-*N*-phenyl-2-naphthalenecarbohydrazide (I).

The molecular structure of (I), C_18_H_14_N_2_O_3_, is illustrated in Fig.1. The bond length and bond angles in (I)are within normal ranges. The dihedral angle between the planes of naphthalene and benzene rings is 2.640 (2)°. Atom O2 is only approximately co-planar with the naphthalene plane and deviates from the benzene plane by 0.788 (2)Å. The maximum atomic deviation (O3) from the naphthalene plane is 1.403 (2)Å.

The mean interplanar distance of 3.339Å between naphthalene rings and 3.357Å between benzene rings suggests that the ligands are engaged in π-π stacking interactions (Fig. 2). The crystal structure of (I) is stabilized by O—H···O and N—H···O hydrogen bonding (Table 1).

### Experimental

Acetic anhydride (6.8 g, 66.8 mmol) and 3-hydroxy-2-naphthalenecarbohydrazide (11.3 g, 56.0 mmol) were added to 120 ml of chloroform with an external ice-water bath. The reaction mixture was slowly warmed to room temperature and stirred for 8 h. After leaving overnight in a refrigerator, the resulting white precipitate was filtered and rinsed with chloroform and diethyl ether. Yield: 95.3%. Melting point: 492 - 496 K.  Calcd. for C_18_H_14_N_2_O_3_: C, 70.58; H, 4.61; N, 9.15%; Found: C, 70.24; H, 4.75; N, 9.02%.

### Refinement

All H atoms were placed in geometrically idealized positions and constrained to ride on their parent atoms (C—H = 0.93%A; N—H = 0.86Å; O—H = 0.82 Å) and *U*_iso_(H) values weren taken to be equal to 1.2 *U*_eq_(C, N) and 1.5U_eq_(O). The hydroxy proton was  located from from difference Fourier maps.  In the absence of significant anomalous scattering effects, Friedel pairs wer merged.

### Crystal data

**Table:**

C_18_H_14_N_2_O_3_	*F*_000_ = 320
*M**_r_* = 306.31	*D*_x_ = 1.434 Mg m^−^^3^
Monoclinic, *P*2_1_	Melting point = 219–223 K
Hall symbol: P 2yb	Mo *K*α radiation λ = 0.71073 Å
*a* = 4.8049 (10) Å	Cell parameters from 4889 reflections
*b* = 5.0231 (10) Å	θ = 3.5–27.5º
*c* = 29.398 (6) Å	µ = 0.10 mm^−^^1^
β = 91.59 (3)º	*T* = 273 (2) K
*V* = 709.3 (2) Å^3^	Platelet, colorless
*Z* = 2	0.35 × 0.24 × 0.14 mm

### Data collection

**Table:**

Rigaku R-AXIS RAPID diffractometer	1798 independent reflections
Radiation source: fine-focus sealed tube	1397 reflections with *I* > 2σ(*I*)
Monochromator: graphite	*R*_int_ = 0.044
*T* = 273(2) K	θ_max_ = 27.5º
ω scans	θ_min_ = 3.5º
Absorption correction: multi-scan(ABSCOR; Higashi,1995)	*h* = −6→6
*T*_min_ = 0.927, *T*_max_ = 0.984	*k* = −6→5
6959 measured reflections	*l* = −38→38

### Refinement

**Table:**

Refinement on *F*^2^	Secondary atom site location: difference Fourier map
Least-squares matrix: full	Hydrogen site location: inferred from neighbouring sites
*R*[*F*^2^ > 2σ(*F*^2^)] = 0.047	H-atom parameters constrained
*wR*(*F*^2^) = 0.112	*w* = 1/[σ^2^(*F*_o_^2^) + (0.0467*P*)^2^ + 0.1714*P*] where *P* = (*F*_o_^2^ + 2*F*_c_^2^)/3
*S* = 1.05	(Δ/σ)_max_ < 0.001
1798 reflections	Δρ_max_ = 0.18 e Å^−^^3^
208 parameters	Δρ_min_ = −0.18 e Å^−^^3^
1 restraint	Extinction correction: none
Primary atom site location: structure-invariant direct methods	

### Special details

**Table:**

Geometry. All e.s.d.'s (except the e.s.d. in the dihedral angle between two l.s. planes) are estimated using the full covariance matrix. The cell e.s.d.'s are taken into account individually in the estimation of e.s.d.'s in distances, angles and torsion angles; correlations between e.s.d.'s in cell parameters are only used when they are defined by crystal symmetry. An approximate (isotropic) treatment of cell e.s.d.'s is used for estimating e.s.d.'s involving l.s. planes.
Refinement. Refinement of *F*^2^ against ALL reflections. The weighted *R*-factor *wR* and goodness of fit *S* are based on *F*^2^, conventional *R*-factors *R* are based on *F*, with *F* set to zero for negative *F*^2^. The threshold expression of *F*^2^ > σ(*F*^2^) is used only for calculating *R*-factors(gt) *etc*. and is not relevant to the choice of reflections for refinement. *R*-factors based on *F*^2^ are statistically about twice as large as those based on *F*, and *R*- factors based on ALL data will be even larger.

### Fractional atomic coordinates and isotropic or equivalent isotropic displacement parameters (Å^2^)

**Table:**

	*x*	*y*	*z*	*U*_iso_*/*U*_eq_	
C1	0.8504 (7)	−0.1340 (8)	0.04316 (11)	0.0580 (10)	
H1A	0.9337	−0.1095	0.0153	0.070*	
C2	0.9282 (8)	0.0197 (9)	0.07930 (11)	0.0644 (11)	
H2A	1.0645	0.1491	0.0759	0.077*	
C3	0.5261 (8)	−0.3656 (8)	0.08826 (12)	0.0646 (11)	
H3A	0.3923	−0.4981	0.0909	0.078*	
C4	0.6474 (8)	−0.3272 (9)	0.04768 (12)	0.0607 (10)	
H4A	0.5941	−0.4312	0.0228	0.073*	
C5	0.5989 (6)	−0.2081 (7)	0.12670 (10)	0.0426 (7)	
C6	0.8049 (6)	−0.0137 (7)	0.12216 (10)	0.0438 (7)	
C7	0.8769 (7)	0.1425 (7)	0.16055 (10)	0.0491 (8)	
H7A	1.0142	0.2715	0.1578	0.059*	
C8	0.4731 (7)	−0.2387 (8)	0.16918 (11)	0.0512 (8)	
H8A	0.3369	−0.3684	0.1724	0.061*	
C9	0.5449 (5)	−0.0842 (6)	0.20558 (9)	0.0362 (6)	
C10	0.7539 (6)	0.1128 (6)	0.20171 (10)	0.0368 (7)	
C11	0.8542 (5)	0.2972 (6)	0.23870 (9)	0.0371 (6)	
C12	0.6027 (5)	0.5289 (6)	0.34255 (9)	0.0366 (7)	
C13	0.6956 (6)	0.7103 (6)	0.38002 (9)	0.0355 (7)	
C14	0.5666 (7)	0.6932 (7)	0.42123 (10)	0.0474 (8)	
H14A	0.4253	0.5694	0.4251	0.057*	
C15	0.9033 (6)	0.8972 (7)	0.37430 (10)	0.0432 (7)	
H15A	0.9915	0.9099	0.3466	0.052*	
C16	0.9798 (7)	1.0656 (7)	0.40988 (12)	0.0536 (9)	
H16A	1.1171	1.1933	0.4058	0.064*	
C17	0.8531 (7)	1.0442 (7)	0.45115 (11)	0.0530 (9)	
H17A	0.9074	1.1549	0.4752	0.064*	
C18	0.6461 (7)	0.8593 (8)	0.45684 (11)	0.0526 (9)	
H18A	0.5595	0.8457	0.4846	0.063*	
N1	0.7200 (5)	0.2895 (6)	0.27768 (8)	0.0417 (6)	
H1B	0.5876	0.1768	0.2812	0.050*	
N2	0.7938 (4)	0.4635 (6)	0.31267 (7)	0.0410 (6)	
H2B	0.9596	0.5276	0.3150	0.049*	
O1	0.4183 (4)	−0.1167 (5)	0.24650 (6)	0.0469 (6)	
H1C	0.3089	−0.2417	0.2447	0.070*	
O2	1.0522 (4)	0.4497 (5)	0.23390 (7)	0.0525 (6)	
O3	0.3630 (4)	0.4405 (6)	0.33929 (7)	0.0533 (6)	

### Atomic displacement parameters (Å^2^)

**Table:**

	*U*^11^	*U*^22^	*U*^33^	*U*^12^	*U*^13^	*U*^23^
C1	0.071 (2)	0.064 (2)	0.0394 (16)	−0.001 (2)	0.0079 (16)	−0.0050 (18)
C2	0.084 (3)	0.065 (2)	0.0450 (18)	−0.024 (2)	0.0158 (17)	−0.0074 (19)
C3	0.071 (2)	0.065 (2)	0.058 (2)	−0.027 (2)	0.0076 (18)	−0.018 (2)
C4	0.067 (2)	0.069 (3)	0.0462 (19)	−0.005 (2)	−0.0014 (17)	−0.0179 (19)
C5	0.0429 (15)	0.0430 (18)	0.0419 (15)	−0.0036 (15)	−0.0031 (12)	−0.0026 (16)
C6	0.0459 (15)	0.0458 (19)	0.0399 (15)	−0.0031 (16)	0.0028 (13)	−0.0007 (16)
C7	0.0528 (18)	0.049 (2)	0.0455 (17)	−0.0197 (17)	0.0061 (14)	−0.0033 (17)
C8	0.0546 (19)	0.0474 (19)	0.0517 (19)	−0.0214 (17)	0.0055 (15)	−0.0060 (17)
C9	0.0339 (13)	0.0352 (16)	0.0394 (14)	−0.0045 (13)	0.0016 (11)	0.0005 (14)
C10	0.0342 (14)	0.0363 (15)	0.0400 (15)	−0.0056 (13)	−0.0002 (12)	−0.0017 (14)
C11	0.0318 (13)	0.0403 (16)	0.0394 (14)	−0.0060 (13)	0.0015 (11)	−0.0003 (14)
C12	0.0287 (13)	0.0412 (16)	0.0400 (15)	−0.0019 (12)	0.0010 (11)	−0.0013 (14)
C13	0.0306 (13)	0.0372 (17)	0.0386 (15)	0.0016 (12)	−0.0010 (11)	−0.0003 (14)
C14	0.0453 (18)	0.051 (2)	0.0463 (18)	−0.0070 (16)	0.0065 (14)	−0.0036 (17)
C15	0.0416 (15)	0.0419 (18)	0.0465 (16)	−0.0018 (15)	0.0074 (12)	0.0006 (16)
C16	0.0467 (18)	0.045 (2)	0.069 (2)	−0.0092 (16)	0.0009 (16)	−0.0090 (19)
C17	0.059 (2)	0.0464 (19)	0.0534 (19)	0.0016 (17)	−0.0068 (16)	−0.0136 (18)
C18	0.0584 (19)	0.057 (2)	0.0426 (16)	−0.0035 (18)	0.0054 (15)	−0.0079 (17)
N1	0.0357 (12)	0.0460 (15)	0.0437 (14)	−0.0133 (12)	0.0058 (10)	−0.0105 (14)
N2	0.0301 (10)	0.0526 (16)	0.0405 (12)	−0.0084 (12)	0.0027 (9)	−0.0116 (13)
O1	0.0505 (12)	0.0464 (13)	0.0443 (11)	−0.0205 (11)	0.0082 (9)	−0.0030 (11)
O2	0.0529 (12)	0.0588 (15)	0.0463 (11)	−0.0268 (12)	0.0101 (9)	−0.0080 (12)
O3	0.0277 (9)	0.0729 (16)	0.0592 (12)	−0.0083 (11)	0.0034 (9)	−0.0176 (14)

### Geometric parameters (Å, °)

**Table:**

C1—C2	1.357 (5)	C11—N1	1.331 (4)
C1—C4	1.385 (5)	C12—O3	1.235 (3)
C1—H1A	0.9300	C12—N2	1.330 (3)
C2—C6	1.417 (4)	C12—C13	1.489 (4)
C2—H2A	0.9300	C13—C14	1.379 (4)
C3—C4	1.356 (5)	C13—C15	1.384 (4)
C3—C5	1.415 (5)	C14—C18	1.384 (4)
C3—H3A	0.9300	C14—H14A	0.9300
C4—H4A	0.9300	C15—C16	1.387 (4)
C5—C6	1.399 (5)	C15—H15A	0.9300
C5—C8	1.411 (4)	C16—C17	1.377 (5)
C6—C7	1.410 (4)	C16—H16A	0.9300
C7—C10	1.369 (4)	C17—C18	1.374 (5)
C7—H7A	0.9300	C17—H17A	0.9300
C8—C9	1.359 (4)	C18—H18A	0.9300
C8—H8A	0.9300	N1—N2	1.388 (3)
C9—O1	1.373 (3)	N1—H1B	0.8600
C9—C10	1.417 (4)	N2—H2B	0.8600
C10—C11	1.497 (4)	O1—H1C	0.8200
C11—O2	1.233 (3)		
			
C2—C1—C4	120.2 (3)	O2—C11—C10	122.4 (3)
C2—C1—H1A	119.9	N1—C11—C10	117.0 (2)
C4—C1—H1A	119.9	O3—C12—N2	121.3 (3)
C1—C2—C6	121.1 (3)	O3—C12—C13	122.5 (3)
C1—C2—H2A	119.5	N2—C12—C13	116.2 (2)
C6—C2—H2A	119.5	C14—C13—C15	119.5 (3)
C4—C3—C5	121.3 (4)	C14—C13—C12	118.6 (3)
C4—C3—H3A	119.3	C15—C13—C12	121.9 (3)
C5—C3—H3A	119.3	C13—C14—C18	120.4 (3)
C3—C4—C1	120.3 (3)	C13—C14—H14A	119.8
C3—C4—H4A	119.8	C18—C14—H14A	119.8
C1—C4—H4A	119.8	C13—C15—C16	119.9 (3)
C6—C5—C8	118.8 (3)	C13—C15—H15A	120.0
C6—C5—C3	118.3 (3)	C16—C15—H15A	120.0
C8—C5—C3	122.9 (3)	C17—C16—C15	120.2 (3)
C5—C6—C7	118.1 (3)	C17—C16—H16A	119.9
C5—C6—C2	118.8 (3)	C15—C16—H16A	119.9
C7—C6—C2	123.1 (3)	C18—C17—C16	119.9 (3)
C10—C7—C6	123.0 (3)	C18—C17—H17A	120.0
C10—C7—H7A	118.5	C16—C17—H17A	120.0
C6—C7—H7A	118.5	C17—C18—C14	120.1 (3)
C9—C8—C5	122.0 (3)	C17—C18—H18A	120.0
C9—C8—H8A	119.0	C14—C18—H18A	120.0
C5—C8—H8A	119.0	C11—N1—N2	120.0 (2)
C8—C9—O1	120.9 (3)	C11—N1—H1B	120.0
C8—C9—C10	120.0 (3)	N2—N1—H1B	120.0
O1—C9—C10	119.1 (2)	C12—N2—N1	118.5 (2)
C7—C10—C9	118.1 (3)	C12—N2—H2B	120.7
C7—C10—C11	115.9 (3)	N1—N2—H2B	120.7
C9—C10—C11	126.0 (2)	C9—O1—H1C	109.5
O2—C11—N1	120.7 (3)		

### Hydrogen-bond geometry (Å, °)

**Table:**

*D*—H···*A*	*D*—H	H···*A*	*D*···*A*	*D*—H···*A*
O1—H1C···O2^i^	0.82	2.00	2.818 (3)	174
N1—H1B···O1	0.86	1.96	2.652 (4)	137
N2—H2B···O3^ii^	0.86	2.09	2.826 (3)	143

Symmetry codes: (i) *x*−1, *y*−1, *z*;  (ii) *x*+1, *y*, *z*.
